# Is working in the emergency department a risk factor for sleep disorders for healthcare workers?

**DOI:** 10.5935/1984-0063.20200051

**Published:** 2021

**Authors:** Ibrahim Çaltekin, Mehmet Hamamcı

**Affiliations:** 1 Yozgat Bozok University Faculty of Medicine, Department of Emergency Medicine - Yozgat - Center - Turkey.; 2 Yozgat Bozok University Faculty of Medicine, Department of Neurology - Yozgat - Center - Turkey.

**Keywords:** Work-Related Stress, Sleep Disorders, Daytime Sleepiness, Insomnia, Sleep Quality, Healthcare Personnel

## Abstract

**Objective:**

This study aims to investigate the relationship between work-related stress and sleep disorders in healthcare personnel working in emergency department and in other departments.

**Material and Methods:**

This cross-sectional study included 34 emergency department healthcare personnel (emergency group [EG]) and 35 healthcare personnel working in other departments (non-emergency group [NEG]) and was conducted between November 10, 2019 and March 1, 2020. All participants were administered the following questionnaires: work-related strain inventory (WRSI), Epworth sleepiness scale (ESS), Berlin questionnaire, insomnia severity index (ISI), Pittsburgh sleep quality index (PSQI), Beck depression inventory (BDI), and Beck anxiety inventory (BAI).

**Results:**

While the mean WRSI score of EG was 39.53±7.77, the mean WRSI score of NEG was 30.06±7.26 (t=5.236, p<0.001). According to PSQI, 79.4% of EG and 57.1% of NEG were found to have poor sleep quality (X^2^=3.938, df=1, *p*=0.047). Median PSQI overall score was 12 (IQR 25th-75th percentiles: 10-14) in EG, and 7 (IQR 25th-75th percentiles: 4-9) in NEG (U=285.5, *p*<0.001). While the mean anxiety score of EG was 13.35±5.70, the mean anxiety score of NEG was 9.06±6.00 (t=3.046, *p*=0.003). Median depression score was 12 (IQR 25th-75th percentiles: 10-16) in EG, and was 8 (IQR 25th-75th percentiles: 4-12) in NEG (U=354, *p*=0.004). A signiﬁcant positive correlation was found between work-related strain scores and sleep quality, sleepiness, and insomnia severity scores (r=0.541, *p*<0.001; r=0.310, *p*=0.010; r=0.357, *p*=0.004; respectively).

**Conclusion:**

It was determined that healthcare personnel working in the emergency department were at higher risk of developing sleep disorders compared to healthcare personnel working in other departments and that there was a signiﬁcant relationship between sleep disorders and work-related stress.

## INTRODUCTION

Work-related stress, defined as an individual’s psychological responses to stressors in their environment, emerges in job conditions with high work demand and generally low social support^1^^,^^2^. Work-related stress is observed in occupations related to intense and arduous conditions. The health field is considered an environment where more work stress is experienced than other occupational fields due to heavy workload, care for severe and terminal patients, and providing emotional support to patients and their relatives when necessary. In studies conducted with healthcare professionals, stressors in the workplace have been found to negatively affect their physical and mental health and job satisfaction^3^^-^^5^.

One study on emergency station workers found that they experienced stress when working due to risk of contracting infection, limited personnel and materials, inability to communicate with colleagues, fear of making mistakes, and vital decisions that need to made quickly^6^. However, exposure to violence is unfortunately a common risk among emergency healthcare workers^7^^,^^8^. People who are exposed to violence are at increased risk of anxiety, emotional exhaustion, desensitization, and burnout syndrome^9^^,^^10^. Emergency care workers who experience posttraumatic stress disorder due to violence experience feelings of fear, terror, and helplessness^11^. Therefore, while job-related stress is a widespread condition among healthcare workers, it is especially serious among emergency department personnel^12^^-^^15^.

The association between sleep disorders and stress, anxiety, and depression has been known for a long time^16^^-^^18^. Significant correlation between sleep disorders and job-related stress has also been reported^19^. People with decreased sleep time and poor sleep quality exhibit both physical and psychological effects^20^. As a result, sleep disorders of healthcare professionals not only affect their health, but also directly affect the health of the patients receiving services^21^.

In light of all of this information, the null hypothesis of the study was that the healthcare personnel working in the emergency department would not have high prevalence of sleeping disorders and work-related stress, compared to personnel working in other departments. In order to test this hypothesis, questionnaires related to work-related stress, anxiety, depression, sleep quality, sleepiness, insomnia, and sleep apnea syndrome were applied to healthcare personnel working in the emergency room and other departments.

## MATERIAL AND METHODS

### Design

This cross-sectional study was conducted in Yozgat Bozok University Hospital between November 10, 2019 and March 1, 2020, and included 34 healthcare personnel working in emergency department and 35 healthcare personnel working in other departments. The study obtained approval from the university’s ethics committee before its start (Protocol: 2017-KAEK- 189_2019.10.16_08). Informed written consent was obtained from all participants. The study was conducted in accordance with the principles of the Declaration of Helsinki.

### Study groups

Healthcare professionals who volunteered to participate in the study consisted of patient care personnel, secretaries, nurses, and doctors. Study groups were divided as follows:

Emergency group (EG): healthcare personnel who worked in the emergency department.

Non-emergency group (NEG): healthcare personnel who worked in departments other than the emergency department (internal medicine: clinics of cardiology, pediatric, radiology, neurology; surgery clinics: outpatient surgery, gynecology and obstetrics, general surgery; intensive care unit: coronary intensive care unit, anesthesia intensive care unit).

Since there were no personnel over age 45 working in the ED, in order to prevent age difference between the groups, personnel over age 45 working in order departments were not included in the study. Therefore, age range of our participants was between 18 to 45 years. All of the emergency personnel who gave their consent to participate in the study and met inclusion criteria for participation, and those working in other departments who were younger than 45 years of age and provided their consent to participate in the study were randomly recruited. Participants who worked in their department for less than one year, who had alcohol-substance and caffeine addiction, those with chronic systemic, psychological and endocrine diseases, and women who were pregnant or breastfeeding were excluded from the study. All participants completed a sociodemographic and clinical data forms. Height and weight were measured and body mass index (BMI) was calculated. Staff of both emergency department and other departments were administered the following questionnaires: work-related strain inventory (WRSI), Epworth sleepiness scale (ESS), Berlin questionnaire, insomnia severity index (ISI), Pittsburgh sleep quality index (PSQI), Beck depression inventory (BDI), and Beck anxiety inventory (BAI).

Two other groups consisting of shift workers and non-shift workers were also formed in order to evaluate the effects of shift work on sleep. Working hours outside of 8 a.m. - 5 p.m. were considered shift work.

### Evaluation tools

#### Data collection form

The data collection form was prepared by the researchers according to scientific sources for study purposes. The form was used to collect data including age, gender, marital status, education level, department they worked at and how long they have worked there, place of residence, level of income, habits, medical history, and history of chronic diseases.

#### Work-related strain inventory (WRSI)

WRSI was designed to measure work-related strain in the work environment. The inventory was first introduced by Revicki et al.^22^ and is an 18-item 4-point Likert scale with responses ranging from does not apply to me (1) to does apply to me (4). Minimum score is 18 and maximum score is 72. Increased score indicates greater work-related stress. In a study conducted by Revicki et al.^23^ predicted general psychological distress with a coefficient alpha of 0.9 and this measure was validated on emergency medicine workers.

#### Beck depression inventory (BDI)

BDI is a 21-item scoring method that was first developed by Beck et al.^24^ to evaluate depressive symptoms. Each item consists of 4 responses ranging from 0 (i.e., “I don’t feel like a failure”) to 3 (i.e., “I feel I am a complete failure as a person”) to evaluate the severity of depression symptoms. Minimum score is 0 and maximum score is 63. Score of 0-9 indicates normal or minimal depression, 10-16 mild depression, 17-29 moderate depression, and 30-63 severe depression.

#### Beck anxiety inventory (BAI)

BAI was developed by Beck et al.^25^ in order to measure the extent of anxiety symptoms and consists of 21 items to determine the autonomic, neurophysiologic, panic, and subjective characteristics of anxiety. Patients rate each symptom from 0 (none) to 3 (severe) according to severity. Minimum score is 0 and maximum score is 63. Increased score indicates higher level of anxiety. Score of 0-7 indicates minimal anxiety, 8-15 mild anxiety, 16-25 moderate anxiety, and 26-63 severe anxiety.

#### Pittsburgh sleep quality index (PSQI)

PSQI is a scale developed by Buysse et al.^26^ in order to provide a standardized, reliable, and valid measure of sleep quality. The scale consists of 18 items comprising of 7 constructs. These constructs include subjective sleep quality, sleep latency, sleep duration, habitual sleep efficiency, sleep disturbances, use of sleeping medication, and daytime dysfunction. Each item is scored from 0-3 and total score ranges from 0 to 21. Total score of 5 and higher indicates clinically poor sleep quality.

#### Epworth sleepiness scale (ESS)

ESS is an 8-item scale developed by Johns et al.^27^ in order to assess daytime sleepiness. Each item is scored from 0-3 and total score ranges between 0 to 24. Higher total score of 10 indicates excessive daytime sleepiness (EDS).

#### Berlin questionnaire

The Berlin questionnaire was developed by Netzer et al.^28^ for screening of obstructive sleep apnea syndrome (OSAS) and consists of 10 items divided into three categories: snoring and breathing symptoms, fatigue/daytime sleepiness, and hypertension/BMI. Each category is evaluated separately; positive results in two or more categories is considered high risk for OSAS.

#### Insomnia severity index (ISI)

ISI was developed by Morin et al.^29^ to assess the effect, severity, and nature of insomnia. Maximum score ranges between 0-28. Score between 0-7 indicates absence of clinically significant insomnia, while score of 8-14 mild, 15-21 moderate, and 22-28 clinically severe insomnia.

### Statistical analysis

Statistical analysis was performed using the SPSS® 22.0 (Statistical Package for Social Sciences, IBM Inc., Chicago, IL, USA) package program. Data distribution was assessed with visual (histogram) and analytical (Kolmogrov-Simirnof) tests. Student’s t-test and Mann- Whitney U tests were applied according to whether or not data was normally distributed. Categorical data were evaluated with Chi-square test. Measurable numerical data were expressed as mean ± standard deviation, categorical data were expressed as number (n, %), and data without normal distribution were expressed as median (minimum-maximum). Spearman’s rho test was used to evaluate correlation of the data. *P* value of less than 0.05 was considered statistically significant.

## RESULTS

Sociodemographic data and statistical analyses of emergency group and non-emergency group are presented in Table 1. As demonstrated in Table 1, there was non-significant difference between the healthcare workers according to age (33.76±6.08 vs. 32.43±5.54) or gender (t=0.955, *p*=0.343; X^2^=0.703, df=1, *p*=0.402; respectively). While 85.3% of EG were shift workers, this rate was 60% in NEG (X^2^=5.53, df=1, *p*=0.019). Furthermore, when all personnel were evaluated, 12% had obesity (BMI>30) and 52% were above normal body weight (BMI>25).

**Table 1:** Sociodemographic and clinical characteristics of the groups.

		Emergency group (n=34)	Non-emergency group (n=35)	p value
Age, mean ± SD		33.76±6.08	32.43±5.54	t=0.955, p=0.343
Gender, n (%)	Female	16 (47.1%)	20 (57.1%)	X^2^=0.703, p=0.402
	Male	18 (52.9%)	15 (42.9%)	
BMI, mean ± SD		25.66±3.33	25.09±4.15	t=0.630, p=0.531
Years of employment, mean ± SD		9.26±5.00	8.71±4.83	t=0.465, p=0.644
Education level, n (%)	High school	6 (17.6%)	7 (20%)	X^2^ =0.963 p=0.810
	Graduate	19 (55.9%)	19 (54.3%)	
	Postgraduate	3 (8.8%)	5 (14.3%)	
	Doctorate	6 (17.6%)	4 (11.4%)	
Marital status, n (%)	Married	23 (67.6%)	21 (60%)	X^2^ =0.437, p=0.509
	Single	11 (32.4%)	14 (40%)	
Smoking, n (%)	Non-smoker	18 (52.9%)	26 (74.3%)	X^2^ =3.401, p=0.065
	Smoker	16 (47.1%)	9 (25.7%)	
Shift-work, n (%)	Normal hours	5 (14.7%)	14 (40%)	X^2^=5.53, p=0.019
	Shift worker	29 (85.3%)	21 (60%)	

SD: Standard deviation; BMI: Body massindex; t: Student t-test; X^2^ = Chi-squaretest.

Comparison of PSQI overall and subscale scores of the two groups are presented in Table 2. According to PSQI scores, 79.4% of EG and 57.1% of NEG were found to have poor sleep quality (X^2^=3.938, df=1, *p*=0.047). In addition, median PSQI overall score was 12 (IQR 25th- 75th percentiles: 10-14) in EG, and 7 (IQR 25th-75th percentiles: 4-9) in NEG (U=285.5, *p*<0.001).

**Table 2:** Pittsburgh sleep quality index scores of the groups.

PSQI Scores, median (25th-75th percentiles)		Emergency group (n=34)	Non-emergency group (n=35)	p-value
PSQI Total		12 (10-14)	7 (4-9)	U=285.5, p<0.001
Subjective Sleep Quality		2 (1-2)	1 (1-2)	U=427, p=0.035
Sleep Latency		3 (2-3)	2 (1-3)	U=406.5, p=0.016
Sleep Duration		1 (1-2)	1 (0-1)	U=409, p=0.015
Habitual Sleep Efficiency		1 (1-2)	1 (0-1)	U=385.5, p=0.008
Sleep Disturbances		2 (1-2)	1 (1-2)	U=328, p<0.001
Daytime Dysfunction		3 (2-3)	1 (0-2)	U=231, p<0.001
Use of Sleeping Medication		0 (0-0)	0 (0-0)	U=542.5, p=0.074
PSQI Total ≥ 5	Yes	27 (79.4%)	20 (57.1%)	X^2^=3.938, p=0.047
	No	7 (20.6%)	15 (42.9%)	

PSQI: Pittsburgh sleep quality index; U: Mann-Whitney U test; X^2^ = Chi-square test.

WRSI, BDI, BAI, ESS, ISI, and Berlin Questionnaire scores of the two groups are presented in Table 3. While the mean WRSI score of EG was 39.53±7.77, the mean WRSI score of NEG was 30.06±7.26 (t=5.236, *p*<0.001). While the mean anxiety score of EG was 13.35±5.70, the mean anxiety score of NEG was 9.06±6.00 (t=3.046, *p*=0.003). Median depression score was 12 (IQR 25th-75th percentiles: 10-16) in EG, and was 8 (IQR 25th-75th percentiles: 4-12) in NEG (U=354, *p*=0.004). EG (12.15±4.18) had higher ISI scores than NEG (9.09±4.64) (t=2.876, *p*=0.005). Median ESS score was 13 (IQR 25th-75th percentiles: 8-18) in EG and 9 (IQR 25th-75th percentiles: 7-15) in NEG (U=429.5, *p*=0.046). In addition, according to ISI scores, 88.2% of EG and 63.5% of NEG had mild or more severe insomnia (X^2^=4.911, df=1, *p*=0.027). There was no statistically significant difference between the two groups according to OSAS risk (X^2^=0.134, df=1, *p*=0.714). BDI and BAI scores of the emergency and non-emergency group are presented in Table 4. According to BDI score, 53% of the EG group had mild depression and 23.5% had moderate depression, while in the NEG group, 31.4% had mild depression and 8.6% moderate depression. According to BAI score, 47.1% of the EG group had mild anxiety, 35.3% moderate anxiety, and 2.9% severe anxiety, whereas in the NEG group, 37.1% had mild anxiety and 17.1% had moderate anxiety. None of the participants in the NEG group had severe anxiety according to BAI scores.

**Table 3:** WRSI, BDI, BAI, ESS, ISI, and Berlin questionnaire scores of the two groups.

		Emergency group (n=34)	Non-emergency group (n=35)	*p*-value
WRSI, mean ± SD		39.53±7.77	30.06±7.26	t=5.236, *p*<0.001
BAI, mean ± SD		13.35±5.70	9.06±6.00	t=3.046, *p*=0.003
BDI, median (25th-75th percentiles)		12 (10-16)	8 (4-12)	U=354, *p*=0.004
ISI, mean ± SD		12.15±4.18	9.09±4.64	t=2.876, *p*=0.005
ESS, median (25th-75th percentiles)		13 (8-18)	9 (7-15)	U=429.5, *p*=0.046
OSAS Risk, n (%)	Low risk	27 (79.4%)	29 (82.9%)	X^2^=0.134, *p*=0.714
	High risk	7 (21.6%)	6 (17.1%)	
ESS Total > 10, n (%)	Yes	20 (58.8%)	10 (28.6%)	X^2^=6.423, *p*=0.011
	No	14 (41.2%)	25 (71.4%)	
ISI > 7, n (%)	Yes	30 (88.2%)	23 (65.3%)	X^2^=4.911, *p*=0.027
	No	4	12	

WRSI: Work-related strain inventory; BAI: Beck anxiety inventory; BDI: Beck depression inventory; ISI: Insomnia severity index; ESS: Epworth sleepiness scale; OSAS: Obstructive sleep apnea syndrome; t: Student t-test, U: Mann-Whitney U test, X^2^ = Chi-square test.

**Table 4:** BDI and BAI scores of the emergency and non-emergency group.

BDI groups	BDI scores	Emergency group (n=34)	Non-emergency group (n=35)
	n	n (%)	n (%)
Normal or minimal	0-9	8 (23.5%)	21 (60%)
Mild	10-16	18 (53%)	11 (31.4%)
Moderate	17-29	8 (23.5%)	3 (8.6%)
Severe	30-63	-	-
**BAI groups**	**BAI scores**	**Emergency group (n=34)**	**Non-emergency group (n=35)**
	**n**	**n (%)**	**n (%)**
Normal or minimal	0-7	5 (14.7%)	16 (45.7%)
Mild	8-15	16 (47.1%)	13 (37.1)
Moderate	16-25	12 (35.3%)	6 (17.1%)
Severe	26-63	1 (2.9%)	-

BDI: Beck depression inventory; BAI: Beck anxiety inventory.

Correlation analyses of sleep scales and work-related strain, length of work, and psychological characteristics are shown in Table 5. As shown in Table 5, in EG a significant correlation was observed between work-related strain scores and sleep quality total score, daytime sleepiness scale, and insomnia severity index scores (r=0.541, *p*<0.001; r=0.310, *p*=0.010; r=0.357, *p*=0.004; respectively).

**Table 5:** Correlation analysis of sleep scale scores and WRSI, length of employment, and psychological characteristics (n=34).

Parameters		PSQI total	ESS total	ISI
WRSI	r_s_	0.541	0.310	0.357
	*p*-value	<0.001	0.010	0.004
Years of employment	r_s_	0.151	0.186	0.090
	*p*-value	0.215	0.126	0.464
BAI	r_s_	0.507	0.496	0.430
	*p*-value	<0.001	<0.001	<0.001
BDI	r_s_	0.578	0.467	0.541
	*p*-value	<0.001	<0.001	<0.001

PSQI: Pittsburgh sleep quality index; WRSI: Work-related strain inventory; ESS: Epworth sleepiness scale; ISI: Insomnia severity index; BAI: Beck anxiety inventory; BDI: Beck depression inventory. Top row indicates correlation coefficients, bottom row indicates p-values.

Evaluation of sleep parameters of shift workers and non-shift workers is shown in Table 6. It was determined that shift workers had poorer sleep quality compared to those who worked normal hours. Median PSQI score was 10 (IQR 25th-75th percentiles: 4-13) in shift workers and 7 (IQR 25th-75th percentiles: 4-11) in non-shift workers (U=313, *p*=0.027).

**Table 6:** Evaluation of sleep parameters of shift workers and non-shift workers.

	Non-shift workers (n:19)	Shift workers (n=50)	*p*-value
PSQI Total, median (25th-75th percentiles)	7 (4-11)	10 (4-13)	U=313, p=0.027
ESS Total, median (25th-75th percentiles)	8 (6-12)	9 (8-18)	U=304.5, p=0.021
ISI, median value (25th-75th percentiles)	9 (6-12)	12 (9-15)	U=287, p=0.011

PSQI: Pittsburgh sleep quality index; ESS: Epworth sleepiness scale; ISI: Insomnia severity index; U: Mann-Whitney U test.

## DISCUSSION

The results of this cross-sectional study indicate that healthcare personnel working in the emergency department have higher work-related stress, and are more affected by anxiety, depression, and sleep disorders. In addition, work-related stress was also correlated with PSQI total scores, ISI, and ESS scores. It was also determined that shift working may be a factor for sleep disorders in healthcare personnel.

Work-related stress may be observed in various occupational groups^30^^,^^31^. Among underlying factors of this condition, working in an intense and stressful job is a factor itself. Nevertheless, since working in the field of health involves human health-related interventions, the aforementioned intense and stressful working conditions are at peak levels in this occupation group^32^^,^^33^. At the same time, another consequence of work-related stress is decreased quality of patient care^34^. Due to the nature of the applying patients and the exposure to violence, emergency department workers experience increased work-related stress^35^. Furthermore, one review, which evaluated the status of work-related stress in emergency department personnel indicated that occupational stress was especially high^36^. Similarly, although our study found that both groups had significantly high WRSI scores, emergency department personnel were found to have relatively higher work-related strain scores than healthcare personnel working in other departments. Similar to our results, Miligi et al.^37^ reported that work-related stress was high in healthcare workers. In addition, a significant relationship between shift work and WRSI in healthcare workers has been reported^38^. This outcome was observed in the shift workers in our study. Collective evaluation of the results of our study together with the literature suggests that emergency work may pose a risk factor for work-related stress in healthcare workers and a mediating factor may be the shift work.

Many factors in work life cause sleep disturbances and problems are generally observed in the age group of working age. Sleep disorders especially emerge in the presence of physical and psychosocial impacts of business life^39^. The ISI scores of the medical staff working in the emergency department were higher than those of the medical staff that working in other departments. Furthermore, according to ISI scores, 88.2% of EG and 63.5% of NEG had mild to more severe insomnia. It has been reported that shift work poses a great risk for insomnia in shift workers^40^. Huang et al.^41^ conducted a study on nurses working in the hospital and reported that high levels of work-related stress and burnout caused insomnia, anxiety, and depression. One recent study indicated that there was a close relationship between insomnia and anxiety and depression^42^. Our study also found that there was a significant relationship between ISI scores and work-related stress. This also suggests that both shift working and the work-related stress caused by working in the emergency department may increase the risk of insomnia.

Our study also found that healthcare personnel working in the emergency department had high rates of daytime sleepiness. It is known that some occupational groups which require working in irregular shifts have increased rates of daytime sleepiness^43^^,^^44^. In fact, circadian rhythm sleep disorders, which include symptoms of insomnia and sleep disorders such as excessive daytime sleepiness, are quite common in healthcare workers^40^. It has also been determined that healthcare workers, especially those groups who have to work in frequent periods, have increased rates of daytime sleepiness. Excessive fatigue, anxiety, and depression were found to be increased in this group, just like daytime sleepiness^45^. It should be noted that these situations all trigger each other. Daytime sleepiness is known to be associated with anxiety and depression levels^17^^,^^46^. The results of our study determined that there was a significant correlation between ESS total scores and work-related strain, anxiety and depression scale scores. This indicates that the healthcare professionals working in the emergency department experience daytime sleepiness, which is very important. Daytime sleepiness is a very negative risk factor for many jobs, which require careful attention, especially driving^47^. In this regard, this points to a great risk for both emergency department staff as well as patients applying to the emergency department. Therefore, this may indicate that health personnel working in the emergency department should be evaluated by a sleep specialist.

According to PSQI scores, it was found that 79.4% of emergency staff had poor sleep quality, compared to 57.1% of staff working in other medical departments. Emergency staff also had significantly worse subjective sleep quality, sleep latency, sleep efficiency, sleep disturbances, and daytime dysfunction compared to the control group. In addition, PSQI total scores were found to be significantly correlated with years of employment and depression and anxiety scores. It was also found that shift working affected sleep quality. Work-related stress was found to impair sleep quality^48^. This may be an indication that working in the emergency department may be an extra stress factor for healthcare professionals and that it may cause poor sleep quality. At the same time, it should not be forgotten that shift work, which forms a basis for reduced sleep quality, is an additional factor.

OSAS, one of the most severe sleep disorders, may cause hypertension, cardiovascular, cerebrovascular, and endocrine disorders. Although OSAS is not a consequence of work-related stress, it may be noted that work-related stress may occur due to OSAS^49^. OSAS is a relatively common sleep disorder; however, it is not sufficiently diagnosed or adequately treated^50^. High risk of OSAS was found in about one of five personnel in both emergency staff and staff of other departments, however there was no significant risk between the groups according to OSAS risk. Obesity was found in 12% of the study participants (BMI>30), while 52% were above normal body weight (BMI>25). According to the literature, OSAS prevalence is estimated to range between 1.2%-28%^51^^-^^54^. Although the Berlin questionnaire is widely used as a screening test for OSAS, it should be noted that there are controversies regarding its diagnostic accuracy. In a meta-analysis, the sensitivity of BQ in detecting OSAS varied between 76% and 84%, while the specificity varied between 38% and 59%^50^. These findings suggest that there is a need for polysomnographic studies on the subject.

Shift working is among the risk factors for sleep disorders^39^. Work outside of routine work hours between 8 a.m. - 5 p.m. is considered shift working. It has been reported that shift workers may have sleep disorders especially due to fatigue^55^. It was determined that 85.3% of emergency personnel and 60% of non-emergency department personnel did shift work. Conway et al.^56^ reported that shift work and work-related stress decreased sleep quality leading to the person’s impaired health, and that these situations triggered each other. The findings of our study indicate that working in the emergency department may pose a risk for sleep disorders, and it was revealed that shift working, which generally occurs in emergency departments, might be one of these causes.

Lack of sleep is a risk factor for occupational accidents. It has been reported that providing sleep training to the emergency medical services (EMS) personnel about sleep management can improve employee health and safety^55^. According to our results, accompanied by relevant literature, taking precautions to reduce work-related stress levels in healthcare workers working in the emergency department may create a significant risk reduction for sleep disorders.

### Limitations

One limitation of our study was the limited number of healthcare personnel due to exclusion criteria and the other limitation of our study was that it was based on self-report questionnaires. In addition, due to the lack of personnel over the age of 45 in our emergency department, healthcare workers over 45 years of age in departments other than the emergency department were not included in our study in order to prevent age-related differences between the groups.

For this reason, our study evaluates young adult healthcare workers and the fact that more advanced age was not evaluated was another limitation. Healthcare professionals of various groups who were in communication with patients and exposed to the same stress in the same hospital environment were included in our study. This situation may be considered as a strength of the study as it includes a holistic approach to healthcare workers, but may also be considered as a limitation as it includes healthcare workers who do not directly impact the patient’s health. In addition, the fact that different professions in the field of health were not evaluated separately may also be considered a limitation. Lack of data regarding shift duration, number of consecutive shifts, and night shift work was also a limitation. Lastly, absence of polysomnography, wrist actigraphy monitoring, and sleep diary data was also a limitation of the study.

## CONCLUSION

In conclusion, the results of our study demonstrate that healthcare personnel working in the emergency department have increased levels work-related stress and sleep disorders.

Taking precautions to reduce work-related stress may ensure reduced risk of sleep disorders in emergency department personnel. Furthermore, it may be beneficial to provide different and flexible working conditions to healthcare professionals determined to have sleep disorders as a result of shift work, another work-related stress factor, which leads to increase in sleep disturbances. Improvements in this issue may improve the health of both employees and patients. However, as far as we know, this is the first study to evaluate the relationship between work-related stress and sleep disorders in emergency department healthcare personnel, it should be noted that further large-scale studies on this subject are necessary to raise awareness and shed light on this topic. We also believe that there is a need for further studies including data related to shift duration, number of consecutive shifts, and night work are needed.
